# Increase in fertility following coal and oil power plant retirements in California

**DOI:** 10.1186/s12940-018-0388-8

**Published:** 2018-05-02

**Authors:** Joan A. Casey, Alison Gemmill, Deborah Karasek, Elizabeth L. Ogburn, Dana E. Goin, Rachel Morello-Frosch

**Affiliations:** 1 Division of Environmental Health Sciences, University of California, Berkeley School of Public Health, 13B University Hall, Berkeley, CA 94729 USA; 2 Program in Public Health, Department of Family, Population and Preventive Medicine, Stony Brook University, HSC, Level 3, Room 071, Stony Brook, NY 11794-8338 USA; 3 Preterm Birth Initiative, University of California, San Francisco, CA, 550 16th Street, San Francisco, CA 94158 USA; 4 Department of Biostatistics, Johns Hopkins University, 615 N. Wolfe Street, Room E3620, Baltimore, MD 21205 USA; 5 Division of Epidemiology, University of California, Berkeley School of Public Health, 50 University Hall, Berkeley, CA 94729 USA; 6 Department of Environmental Science, Policy & Management and the University of California, Berkeley School of Public Health, 130 Mulford Hall, Berkeley, Berkeley, CA 94720 USA

**Keywords:** Fertility, Live birth, Birth certificates, Coal, Power plants, California, Environmental epidemiology, Natural experiment

## Abstract

**Background:**

Few studies have explored the relationship between air pollution and fertility. We used a natural experiment in California when coal and oil power plants retired to estimate associations with nearby fertility rates.

**Methods:**

We used a difference-in-differences negative binomial model on the incident rate ratio scale to analyze the change in annual fertility rates among California mothers living within 0-5 km and 5-10 km of 8 retired power plants between 2001 and 2011. The difference-in-differences method isolates the portion of the pre- versus post-retirement contrast in the 0-5 km and 5-10 km bins, respectively, that is due to retirement rather than secular trends. We controlled for secular trends with mothers living 10-20 km away. Adjusted models included fixed effects for power plant, proportion Hispanic, Black, high school educated, and aged > 30 years mothers, and neighborhood poverty and educational attainment.

**Results:**

Analyses included 58,909 live births. In adjusted models, we estimated that after power plant retirement annual fertility rates per 1000 women aged 15–44 years increased by 8 births within 5 km and 2 births within 5-10 km of power plants, corresponding to incident rate ratios of 1.2 (95% CI: 1.1–1.4) and 1.1 (95% CI: 1.0–1.2), respectively. We implemented a negative exposure control by randomly selecting power plants that did not retire and repeating our analysis with those locations using the retirement dates from original 8 power plants. There was no association, suggesting that statewide temporal trends may not account for results.

**Conclusions:**

Fertility rates among nearby populations appeared to increase after coal and oil power plant retirements. Our study design limited the possibility that our findings resulted from temporal trends or changes in population composition. These results require confirmation in other populations, given known methodological limitations of ecologic study designs.

**Electronic supplementary material:**

The online version of this article (10.1186/s12940-018-0388-8) contains supplementary material, which is available to authorized users.

## Introduction

Oil and coal power plants can emit significant air pollution, including particulate matter (PM), sulfur dioxide (SO_2_), benzene, lead, mercury, and other known health hazards [1]. The amount of emitted pollution varies, however, based on fuel usage and type, control technologies (e.g., scrubbers), stack heights, and meteorological conditions [2]. While populations living closest to power plants experience higher levels of primary air pollutants, substantial exposure to secondary particulate matter can occur up to 500 km away [3].

Residential proximity to power plants has been linked to health outcomes such as cardiovascular and respiratory disease [4] and adverse birth outcomes [5, 6]. Coal and oil plants, especially older and less efficient ones, typically emit more pollution than those run on natural gas [7]. Between 2007 and 2016 coal-fired electricity generation dropped by 40% in the U.S [8]. Future coal and oil power plant retirements–driven by energy efficiency, abundant and cheap natural gas, and legislation related to climate change–may benefit the health of those living nearby.

Another possible result of power plant retirement is improved fertility among nearby populations. Some evidence suggests links between environmental factors and infertility [9], but few studies have investigated the role of air pollution on reproductive outcomes such as live birth rates, infertility, and miscarriage [10]. Three studies have reported associations between traffic-related air pollution and longer time-to-pregnancy, infertility, and spontaneous abortion [11–13]. Fewer than expected miscarriages and stillbirths were reported after a coal plant retirement in Croatia [14].

Only Nieuwenhuijsen et al. (2014) have examined the relationship between air pollution and fertility rates in a general population. They reported an association between higher levels of traffic-related pollution and coarse fraction particulate matter (PM_2.5–10_) in Barcelona and reduced fertility [15]. The components of air pollution differ between that generated by traffic and power plants, however, and the authors noted the potential for residual confounding due to their ecological design.

Research suggests that residential proximity to industrial facilities, including power plants, is also associated with adverse mental health outcomes, including stress and depression, due to perceptions of neighborhood disorder and feelings of personal powerlessness [16, 17]. This may have implications for fertility based on evidence linking higher levels of stress with difficulty conceiving [18].

We sought to expand this limited research by capitalizing on a natural experiment when 8 coal and oil power plants retired in California between 2001 and 2011. Natural experiments enable researchers to investigate the impact of modifiable factors or interventions on health outcomes by exploiting changes in exposure that are difficult to manipulate experimentally [19]. Natural experiments effectively randomize exposure, reducing the threat of residual confounding inherent in observational studies [20]. We implemented a quasi-experimental difference-in-differences approach to estimate the association between coal and oil power plant retirements and nearby fertility rates.

## Methods

### Study population

We obtained birth certificate data from January 1, 2001 to December 31, 2011 from the California Department of Public Health. We abstracted infant sex and birth date, maternal address, age, date of last menstrual period (LMP), race/ethnicity, and educational attainment from birth certificates. After exclusions (Additional file 1: Figure S1, available in Supplement), 558,308 live births took place within 20 km of a retiring coal or oil power plant during the study period.

### Power plant exposure

**Fig. 1:**
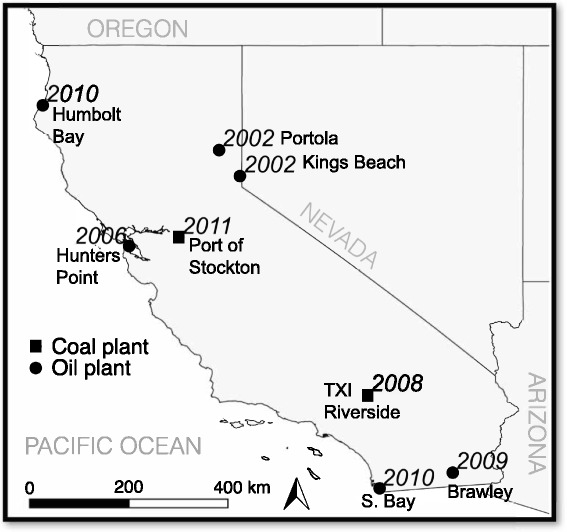
Location and names of 8 coal and oil power plants in California with year of retirement

We downloaded data on coal and oil power plants in California from the U.S. Energy Information Agency (EIA) [8], the U.S. Environmental Protection Agency (EPA) Air Markets Program [21], and the California EPA Air Resources Board (CARB) [22]. These data contained plant latitude and longitude, fuel type, start date, retirement date, and monthly fuel consumption. CARB also provided annual NO_x_ emissions data on power plants that retired after 2007. We obtained NO_x_ emissions data on the Hunters Point plant from the EPA Air Markets Program database but were unable to locate emissions data on the two smallest power plants that retired in 2002. We included power plants as exposure areas in the analysis if they used coal or oil as a primary fuel (e.g., bituminous coal, distillate fuel oil, and residual fuel oil) and if they retired between 2001 and 2011. Eight California power plants met these criteria (Fig. 1), 6 oil and 2 coal. Two power plants were eventually converted to burn biomass, but this transition required additional permitting and took over a year, meaning they were not active during the study period. For simplicity, we refer to the date of retirement from coal or oil use as “retirement,” even if the plant later transitioned to a new fuel.

**Fig. 2:**
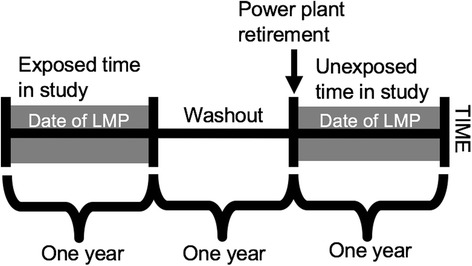
Identification of exposed and unexposed births based on last menstrual period among California mothers, 2001–2011

Based on maternal address at the time of delivery, we used QGIS (qgis.org, QGIS Development Team) to identify births that took place within a 20 km radius of one of the eight power plants. In order to assess a gradient of exposure, we assigned each birth to one of three area bins within the larger 20 km-radius circular area: 0-5 km; 5-10 km; and 10-20 km from a power plant. We used these area bins–0-5 km; 5-10 km; and 10-20 km–and the population of women aged 15–44 years living within them as the units of analysis. Next, we applied temporal restriction criteria. We defined births to mothers with an LMP in the year following power plant retirement as unexposed and births to mothers with an LMP between 1 and 2 years prior to power plant retirement as exposed (Fig. 2). These definitions had the advantage of providing a one-year washout period and of matching exposed and unexposed periods to account for fixed cohort bias, seasonal trends in fertility, and power plant emissions (which peak during the summer months). We anticipated that fertility trends among women living 10-20 km from power plants would be similar to those located ≤10 km in the absence of power plant retirements. Compared to a ≤ 10 km group, the 10-20 km group would experience a smaller change in power plant-related exposure after retirement. Therefore, this 10-20 km group allowed us to control for temporal trends in fertility and isolate changes due to plant retirement.

### Fertility rate

For each of the 8 plants, we estimated the number of women aged 15–44 years living within 20 km before and after retirement using block group level data from the closest, non-overlapping censuses: 2000 U.S. census and 2005–2009, 2006–2010, 2008–2012, 2009–2013, and 2011–2015 American Community Survey (ACS) [23]. For example, the Kings Beach plant retired in December 2002 and we estimated the population before retirement with 2000 census data and the population after retirement with 2005–2009 ACS data. When block groups did not fall entirely in specific area bins, we used geographic-area weighting to allocate the population. We operationalized fertility rate as the number of live births per 1000 women aged 15–44 years within 5 km, 5-10 km, and 10-20 km of power plants before and after retirement.

### Neighborhood data

For our primary analysis, we downloaded block group level data on the number of individuals living below the federal poverty threshold and who attained less than a high school education from the 2000 U.S. census and the ACS [23]. Based on mother’s address at the time of birth we linked block group data. In sensitivity analyses we used additional years of ACS data as described below. We also obtained address-level 2005–2011 annual foreclosure data across California from CoreLogic (formerly DataQuick), which we assigned based on mother’s census block group and infant’s birth year.

### Statistical analysis

We used a difference-in-differences design [24] to compare fertility rates within 20 km of power plants before and after retirement. The difference-in-differences method isolates the portion of the pre- versus post-retirement contrast in the 0-5 km and 5-10 km bins, respectively, that is due to retirement rather than secular trends [24]. We used a negative binomial model to estimate the association between power plant retirements and annual fertility rates on the incident rate ratio scale [25]. Under the assumption that secular trends are parallel in both groups and that the negative binomial was correctly specified, the resulting difference-in-differences estimator corresponds to log-fertility incidence rate ratio. We obtained the difference-in-differences estimates from the product interaction term of two indicator variables: one for the area bin (i.e., 0-5 km or 5-10 km, with 10-20 km as the reference) and one for exposure (i.e., post-retirement, with pre-retirement as the reference). We hypothesized that we would observe a difference-in-differences estimators greater than 1, representing increased fertility rates in the communities 0-5 km and 5-10 km from the power plants after retirement. We identified predictors of fertility a priori [26–28] and controlled for them to increase precision of our model [29, 30]. Adjusted models included fixed effects for power plant, and the proportion births to mothers ≥30 years, mothers of Black or Hispanic race/ethnicity, and mothers with ≤ a high school degree in each plant-area bin. They also included census block group poverty and educational attainment to account for neighborhood characteristics. Finally, we used the estimated parameters of the adjusted negative binomial model to calculate the number of births per 1000 women at the mean values of all covariates. Regression analyses were implemented in Stata version 13.1 (StataCorp LP, College Station, TX) and R Statistical Software, version 3.3.2 (R-project.org, R Core Team).

### Sensitivity analyses

We performed several sensitivity analyses. First, to assess if small numbers were driving the observed associations, we repeated the analysis after omitting Kings Beach and Portola plants, the plants with the fewest women of childbearing age living within 20 km. Second, we stratified the overall analyses by primary fuel type: coal or oil. Third, we implemented a negative exposure control [31] by randomly selecting 8 operational (i.e., non-retiring) power plants in California. We repeated the analyses using the geographic location of these operating plants and by randomly assigning to them the retirement dates of the original 8 retired plants. An association with the negative exposure control would have suggested that observed temporal changes in fertility were unrelated to power plant retirement.

Demographic shifts, particularly those related to the Great Recession, a period of substantial economic downturn, and the housing foreclosure crisis that peaked in California in the summer of 2008 [32–34], could have influenced our results. Because block group level ACS data only exists in 5-year windows, we included the annual number of block group level foreclosures as an indicator of neighborhood socioeconomic deprivation and re-ran analyses for the years 2005–2011. We also used block group level data [23] prior to and after power plant retirements to explore changes in the number and composition of people living near plants after they retired. We estimated the change in the number of total, non-Hispanic white residents, and women aged 15–44 years, the percent of population living below the federal poverty threshold, and median household income.

Finally, we linked daily PM_2.5_ data from the U.S. Environmental Protection Agency Community Multiscale Air Quality Model (CMAQ) to mothers’ block groups to assess changes in air pollution exposures near retiring power plants. We compared average annual PM_2.5_ levels in the 1–2 years prior to the year after power plant retirements.

## Results

Eight California power plants–2 coal and 6 oil–retired between 2001 and 2011 (Additional file 1: Table S1 available in supplement). The U.S. EIA reported that the amount of coal used for electricity generation in California declined over the study period from nearly 1 million short tons per year in 2000 to 539,439 short tons in 2012 [35]. The amount of distillate fuel oil and residual fuel oil also declined (Additional file 1: eFigure S2 available in supplement). Average nitrogen oxide (NOx) emissions from the 8 power plants in our analysis fell from 177 tons in the year prior to retirements to 4 tons per year in the year post-retirement [21, 22]. CMAQ data also suggested larger reductions in PM_2.5_ after power plant retirement in the < 5 km area bin (median pre vs. post: 12.8 μg/m^3^ vs. 10.8 μg/m^3^) compared to the 10-20 km area bin (median pre vs. post: 10.8 μg/m^3^ vs. 10.2 μg/m^3^) (Additional file 1: eTable S2).

The main analysis included 58,909 live births. We observed an average annual fertility rate of 45 births per 1000 women aged 15–44 years (SD = 24). Temporal trends in maternal and neighborhood characteristics appeared similar by area bin (Table 1). We did note, however, a greater increase in the proportion of births to Hispanic and non-Hispanic Black mothers in the < 5 km bin compared to the 5-10 km and 10-20 km bins. The number of foreclosures increased across all three exposure buffers in the unexposed period, reflecting temporal changes in the housing market.

**Table 1:** Maternal and neighborhood characteristics of births occurring in California before and after power plant retirement by area bin around power plants between 2001 and 2011

	1–2 years before retirement			1 year after retirement		
	Area bin around power plant			Area bin around power plant		
Characteristic	0-5 km	5-10 km	10-20 km	0-5 km	5-10 km	10-20 km
N (%)	4329 (15)	8069 (28)	16,581 (57)	4668 (16)	8400 (28)	16,862 (56)
Fertility rate (live births per 1000 women aged 15–44 years per year), median (IQR)	38 (24–63)	32 (28–50)	35 (20–62)	42 (33–74)	35 (30–57)	29 (23–64)
Maternal characteristics						
Percent > 30 years of age, median (IQR)	27 (16–38)	37 (31–84)	38 (31–49)	31 (23–52)	35 (28–54)	40 (20–50)
Percent Hispanic, median (IQR)	50 (26–70)	38 (16–57)	37 (19–48)	58 (28–81)	37 (7–61)	36 (25–46)
Percent non-Hispanic Black, median (IQR)	3.3 (0.1–6.6)	2.6 (0.1–7.5)	3.8 (0.7–10.7)	7.2 (2.3–11.4)	2.3 (0.1–9.1)	3.7 (0.1–12.3)
Percent with high school degree or less, median (IQR)	64 (50–85)	48 (14–68)	44 (31–54)	64 (48–77)	56 (33–87)	42 (33–54)
Neighborhood characteristics^a^						
Percent living below federal poverty threshold, median (IQR)	20 (15–26)	13 (7–22)	15 (10–19)	18 (16–23)	14 (8–14)	16 (10–20)
Percent with < high school diploma or equivalent, median (IQR)	19 (12–21)	16 (10–19)	12 (6–14)	18 (13–20)	14 (12–19)	12 (6–14)
Annual foreclosures, median (IQR)	1 (0–7)	3 (0–21)	2 (0–18)	3 (0–5)	6 (0–39)	1 (1–50)

*Note*. IQR, interquartile range

^a^ Neighborhood characteristics assigned based on mother’s block group of residence at time of birth; poverty and educational attainment from the 2000 US Census and the 2005–2009 American Community Survey; California foreclosure data from CoreLogic (formerly DataQuick)

**Fig. 3:**
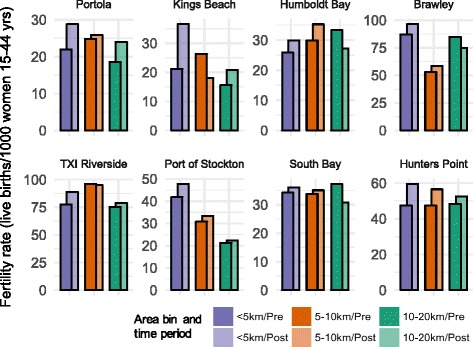
Unadjusted annual fertility rate (number of live births per 1000 women aged 15–44 years) by area bin and power plant before and after power plant retirement. Purple bars represent the closest area bin (< 5 km), orange bars the 5-10 km bin, and green bars the 10-20 km bin. Within area bin, the more saturated bars denote the pre-period and the less saturated bars the post-period. The power plants are ordered by the average number of women aged 15–44 years of age living within 20 km, ranging from 820 women near the Portola plant to 464,599 within 20 km of the Hunters Point plant

Unadjusted fertility rates varied by power plant and area bin (Fig. 3). We expected minimal confounding due to the difference-in-differences design, and a comparison of point estimates between the unadjusted and adjusted models suggested that the included covariates did not confound the association (Table 2). In the < 5 km area bin, the incident rate ratio (IRR) from the adjusted model was 1.2 (95% CI: 1.1–1.4); this corresponds to an annual fertility rate of 51 births per 1000 women aged 15–44 years post-retirement and 43 births per 1000 women aged 15–44 years pre-retirement. In the 5-10 km buffer, the IRR was 1.1 (95% CI: 1.0–1.2), corresponding to an annual fertility rate of 47 births per 1000 women aged 15–44 years post-retirement and 45 births per 1000 women aged 15–44 years pre-retirement. We observed little change in annual fertility rates in the 10-20 km area bin; the annual fertility rate in the pre-period was 41 (95% CI: 26–57) compared to 42 (95% CI: 25–59) live births per 1000 women aged 15–44 years in the post-period.

**Table 2:** Change in the annual fertility rate (live births per 1000 women aged 15–44 years) from 2001 to 2011 in California after 8 coal and oil power plant retirements

	Live births, N (%)	Annual fertility rate Mean (95% CI)^a^			
		Unadjusted		Adjusted^b^	
		1-2 years before retirement	1 year after retirement	1–2 years before retirement	1 year after retirement
Area bin^c^					
0–5 km	8997 (15.3)	45 (28–61)	53 (36–70)	43 (34–53)	51 (41–61)
5–10 km	16,469 (28.0)	43 (27–58)	45 (29–61)	45 (36–54)	47 (38–56)

^a^ Fertility rate obtained from difference-in-differences negative binomial regression model with robust standard errors

^b^ Estimated at the mean value of covariates: power plant and proportion of births to Hispanic and non-Hispanic black mothers, mothers > 30 years of age, and mothers that attained a high school degree or less, and census block group level poverty (%) and individuals with < high school education (%). Standard errors estimated using the delta method

^c^ 10-20 km area bin served as the comparison population

We completed several robustness checks. When we omitted two power plants with the fewest women of childbearing age living nearby results remained similar (IRR = 1.2 [95% CI: 1.0–1.4], IRR = 1.1 [95% CI: 1.0–1.3] in the 0-5 km and 5-10 km bins, respectively). This suggests small, variable rates did not drive the observed association. We observed little change in adjusted associations when stratifying the overall analysis by primary fuel type, except we no longer observed an association between power plant retirement and fertility rates in the 5-10 km bin for coal power plants (Additional file 1: Table S3). While the 10-20 km bins controlled for secular trends local to each plant, in order to further assess the possible effect of statewide trends on our results we implemented negative exposure control analysis, using power plants that did not retire during the study period. This included biomass, geothermal, hydroelectric, natural gas, oil, and solar plants. When combining the location of these plants and their surrounding births with the retirement dates of the original eight power plants we found no significant changes in fertility rates (IRR = 1.1 [95% CI: 0.8–1.3] and IRR = 1.1 [95% CI: 0.9–1.3] in the 0-5 km and 5-10 km bins, respectively, Additional file 1: Table S4). This result suggested that statewide temporal trends may not have accounted for our results. When we integrated foreclosures occurring in the year of birth in block groups within each bin, estimates remained similar (7 additional births per 1000 women in the 0-5 km bin and 1 additional birth per 1000 women in the 5-10 km bin, Additional file 1: Table S5). Finally, we did not identify any major population shifts between the unexposed and exposed time periods that could explain results (Additional file 1: Table S6).

## Discussion

Our study leveraged a natural experiment of 8 coal and oil power plants retirements and found an association with increased fertility rates nearby. Annual fertility rates increased by 8 births per 1000 women aged 15–44 years in the immediate 5 km radius. We observed a gradient, fertility increased the most within 5 km and to a lesser extent within 5-10 km. Because exposed periods occurred prior to unexposed periods and several plant retirements occurred proximal to the housing foreclosure crisis [32–34] temporal trends could have influenced our results. Therefore, we conducted several sensitivity analyses to assess demographic changes over time among residents living near power plants. We found no evidence that these compositional changes explained our findings.

Prior literature has assessed the association between power plant proximity and birth outcomes [5, 6, 36, 37]. We extended this literature, finding an association between power plant retirements and increased fertility. Combustion of petroleum products releases numerous air pollutants, such as SO_2_, NOx, PM, and trace metals like mercury [38] previously linked to reduced human fertility [11, 15]. Stress and mental health among individuals living near power plants may also have played a role [18].

Several mechanisms could explain our findings of increased fertility in the year following plant retirements. Fecundability (i.e., the probability of conception) may have improved. Even exposure to relatively low levels of PM_2.5_, PM_10_, and ozone has been associated with changes in sperm morphology and increases in sperm containing increased DNA fragmentation [39]. In a Czech study of 1916 couples, a 10 μg/m^3^ increase in PM_2.5_ level was associated with a 22% reduction in fecundability [40]. Air pollution may lead to oxidative stress or endocrine disruption that could explain observed changes in sperm and conception more broadly. In vitro fertilization studies have also demonstrated associations between some–NO_2_, PM_2.5_, and PM_10_–but not all, air pollutants and disrupted fertilization and implantation [41–43].

Prior literature has reported changes of a similar magnitude in fertility associated with air pollution exposures. While traffic and power plant air pollution differ [44], traffic studies provide information about potential mechanisms of action related to increased fertility. Nieuwenhuiisen (2014) found a 13% reduction in census tract fertility rates with IQR increases in PM_2.5–10_ from traffic in Barcelona, Spain during 2011–2012 [15]. A study using data from 116,430 nurses on self-reported infertility (defined as attempting to become pregnant for one year without success) found an 11% increase in the hazard of infertility among women living < 200 m (versus ≥200 m) from a major roadway [11]. Self-reported data indicated that ovulatory disorder and male infertility may have driven these associations [11]. In Texas and Michigan, among 393 couples attempting to conceive, couples were 3% more likely to become pregnant for each additional 200 m they lived away from a major roadway [12]. These changes in fertility may result from endocrine disruption, oxidative stress, or DNA modifications caused by pollutants [10].

A reduction in spontaneous abortion may also help explain our findings. As many as 10–20% of clinically recognized pregnancies end in a pregnancy loss [45], and research has shown that these rates increase following period of stress, such as natural disasters [46]. Our results are consistent with evidence that reports associations between pollutant exposures and increased fetal loss. One study found reduced miscarriage and stillbirth following the temporary closure of a coal plant in Croatia [14]. Two observational studies (350,000 and 750,000 live births, respectively) in New Jersey and Ohio found elevated odds of stillbirth (versus live birth) with increased exposure to ambient SO_2_, carbon monoxide, and PM_2.5_ in the third trimester [47, 48]. In Mongolia, which experiences high levels of air pollution from coal combustion in winter months, researchers found a correlation between ambient PM_2.5_, NO_2_ and SO_2_ levels and fetal death [49].

In addition to changes in air quality, the closure of oil and coal power plants may have changed economic conditions for individuals living nearby and thus affected fertility. For instance, if residential proximity was related to employment at the power plants, closure may have resulted in more job loss among individuals living closer to the power plants (i.e., < 5 km). We do not have data to assess this pathway, but job loss would have likely reduced fertility [50], biasing our results towards the null. In addition, other aspects of power plants could have affected fertility rates. Some studies suggest a link between industrial activities and stress, which have been associated with reduced fertility [16, 18].

We assessed whether compositional changes and economic improvement around power plants after retirement might have explained our results. First, we restricted the unexposed period to the single year after power plant retirement, limiting compositional changes that may have occurred over longer periods of time. Second, using ACS data we found minimal changes in population composition, such as small changes in neighborhood poverty following plant retirements. We also assessed whether changes in the housing market due to the foreclosure crisis affected fertility, unrelated to contemporaneous power plant retirements. Models adjusted for the number of annual foreclosures by area bin did not differ from our original results. This adjustment, however, may have been insufficient if temporal changes in foreclosures differed by area bin pre- and post-retirement [51]. Finally, we used communities located 10-20 km from power plants as controls under the assumption that they might have had similar composition to communities located closer but would experience smaller changes in air pollution due to power plant retirements. The choice of control group may have produced conservative estimates, since pollutants from coal plants can travel over 10 km [1].

Assessment of spatial and temporal exposures has been noted as a methodological concern in studies of air pollution and reproductive health [52]. Our design, utilizing a natural experiment of plant closures, addresses these issues through specification of exposure location and timing. As fertility did not cause the closing of plants, staggered closing dates allowed us to approximate a randomized design using difference-in-differences methods.

Our study had several limitations. Because fertility is governed by both biological and behavioral factors [28, 53], our outcome variable (i.e., the fertility rate) lacks specificity to identify pure biological effects. The estimated benefit of power plant retirement for area-level fertility rate may not apply to individual couples attempting to conceive. In addition, the fertility rate denominator estimate included error. The ACS provides population estimates at the block group level in 5-year windows, not annually, and block groups did not perfectly overlap with our selected area bins. Furthermore, we assumed a constant population density of women 15–44 years across block groups, which also added uncertainty to fertility rate estimates. Several of the plants had a relatively small number of births occur within 20 km, which could have resulted in variable rates and spurious associations. To assess this issue, we repeated analysis after removing the two plants with the fewest women of childbearing age nearby and noted no qualitative change in results. We also lacked air quality measurements at mothers’ homes or places of work meaning we could not account for varying levels of background air pollution or identify a single pollutant as the potential causative agent. However, CMAQ PM_2.5_ data did suggest more reduction in air pollution closer to power plants after retirement. Future analyses could explore whether other sources of air pollution, for example biomass plants or traffic, contribute to changes in fertility. Two of the eight power plants eventually transitioned to burning biomass, which release air pollutants. Such transitions take time. For example, the Port of Stockton coal plant retired in January of 2011 and did not reopen to burn biomass until February of 2014 [54]. We treated these transitions, therefore, as retirements for the purpose of this study where we compared the year after retirement to the period 1–2 years prior. In addition, we assigned exposure based on the residential address on the birth certificate and could not account for exposure accrued from work, travel, or residential mobility during pregnancy. We were unable to assess the association of plant closures with neighborhood perceptions and maternal stress. Finally, an assumption of our difference-in-differences analysis is that secular trends in fertility in the 10-20 km area bin mirror that of the < 5 km and 5-10 km area bins, conditional on measured covariates. Although the demographics of the distance bins may have exhibited some differences over the study period [51], unadjusted and adjusted estimates were similar, suggesting that any differences in the measured covariates over time did not confound our results.

## Conclusion

Our results indicate potential fertility benefits associated with coal and oil power plant retirements in California. These results require confirmation in other populations, given known methodological limitations of ecologic study designs. The two coal-fired generators retired in this study had capacities–54 MW and 24 MW–about 1 order of magnitude lower than the nationwide average coal plant. Therefore, we might expect stronger associations in other locations. The U.S. EIA has identified nearly 7000 MW of coal and oil capacity in 8 states slated for retirement in 2018 alone. The Clean Power Plan [55]–the federal government’s strategy to curb carbon dioxide emissions from electricity generation–and similar climate change policies will also likely lead to more power plant retirements as states strive to meet emissions standards. Our study design limited the possibility that the estimated increase in fertility rates after coal and oil power plant retirements resulted from temporal trends or changes in population composition. Ecologic designs have known methodological limitations, however, so our results require confirmation in other populations. Future plant retirements will afford opportunities to further assess their potential reproductive health benefits.

## Additional file

Additional file 1: **Figure S1.** Flow chart of study population assembly. **Figure S2.** Changes in coal and oil used for electricity generation in California between 2000 and 2012. **Table S1.** Characteristics of the 8 coal and oil power plants that retired between 2001 and 2011. **Table S2.** Change in median and interquartile range PM_2.5_ concentrations by area bin. **Table S3.** Analysis stratified by fuel type for the change in the annual fertility rate between 2001 and 2011in California after 8 coal and oil power plant retirements. **Table S4.** Negative control analysis for the change in the annual fertility rate between 2001 and 2011in California after 8 coal and oil power plant retirements. **Table S5.** Change in the annual fertility rate between 2001–2011in California after 8 coal and oil power plant retirements, further adjusted for annual housing foreclosures. **Table S6.** Demographic changes comparing before and after coal and oil power plant retirements in California, between 2001–2011. (DOCX 379 kb)

## Abbreviations

ACS: American Community Survey
CARB: California Air Resources Board
CMAQ: Community Multiscale Air Quality Model
IRR: incident rate ratio
LMP: last menstrual period
NO_x_: nitrogen oxide
PM: particulate matter
SO_2_: sulfur dioxide
U.S. EIA: U.S. Energy Information Agency
U.S. EPA: U.S. Environmental Protection Agency
