# Supplementary material for: Increase in fertility following coal and oil power plant retirements in California
Source: Environ Health. 2018 May 2;17:44. doi: 10.1186/s12940-018-0388-8 (PMC5932773; doi:10.1186/s12940-018-0388-8)
Supplement: Supplementary file 1 — Figure S1. Flow chart of study population assembly. Figure S2. Changes in coal and oil used for electricity generation in California between 2000 and 2012. Table S1. Characteristics of the 8 coal and oil power plants that retired between 2001 and 2011. Table S2. Change in median and interquartile range PM2.5 concentrations by area bin. Table S3. Analysis stratified by fuel type for the change in the annual fertility rate between 2001 and 2011in California after 8 coal and oil power plant retirements. Table S4. Negative control analysis for the change in the annual fertility rate between 2001 and 2011in California after 8 coal and oil power plant retirements. Table S5. Change in the annual fertility rate between 2001–2011in California after 8 coal and oil power plant retirements, further adjusted for annual housing foreclosures. Table S6. Demographic changes comparing before and after coal and oil power plant retirements in California, between 2001–2011. (DOCX 379 kb) [file 12940_2018_388_MOESM1_ESM.docx]

Additional file

**Figure 1:** Flow chart of study population assembly

**Figure 2:** Changes in coal and oil used for electricity generation in California between 2000-2012. Annotations denote closure dates of the two coal and six oil power plants. These data are available from the U.S. Energy Information Administration at <https://www.eia.gov/electricity/data/eia923/>. *Note*: DFO, distillate fuel oil; RFO, residual fuel oil

The California electricity crisis took place during 2000-2001, ignited by a variety of factors including energy deregulation that enabled market manipulation and extremely hot weather in the summer of 2000 that fueled demand. Plants burned additional residual fuel oil during this period to meet consumer demand. To learn more about the energy crisis see the Public Broadcasting Station (PBS) Frontline episode and accompanying information at <https://www.pbs.org/wgbh/pages/frontline/shows/blackout/california/timeline.html> and JL Sweeney’s book on the topic: Sweeney, J.L., 2013. *California Electricity Crisis*. Hoover Press.

**Table 1**: Characteristics of the 8 coal and oil power plants that retired between 2001-2011

| **Power plant** | **EIA ID** | **Plant #^a^** | **Date opened** | **Date retired^b^** | **Fuel** | **Capacity (MW)** |
| --- | --- | --- | --- | --- | --- | --- |
| TXI Riverside | 50557 | 1 | 12/01/1954 | 03/31/2008 | BIT, PC, NG | 24 |
| Port of Stockton | 54238 | 2 | 12/01/1989 | 01/01/2011 | BIT, RC, NG | 54 |
| Kings Beach | 6518 | 3 | 01/01/1969 | 12/31/2002 | DFO | 3 |
| Portola | 6524 | 4 | 01/01/1965 | 12/31/2002 | DFO | 2 |
| Hunters Point | 247 | 5 | 12/12/1948 | 05/15/2006 | DFO, RFO, NG | 52 |
| Brawley | 383 | 6 | 01/01/1962 | 12/31/2009 | DFO | 12 |
| Humboldt Bay | 246 | 7 | 08/20/1975 | 09/24/2010 | DFO, RFO, NG | 52,^c^ 53  (2 units) |
| South Bay | 310 | 8 | 10/12/1966 | 12/31/2010 | DFO, RFO, NG | 700^d^ |

*Note*. BIT, bituminous coal; DFO, distillate fuel oil; EIA, U.S. Energy Information Administration; MW, megawatt; NG, natural gas; PC, petroleum coke; RC, refined coal; RFO, residual fuel oil.

^a^ Plant numbers correspond to **eFigure2.**

^b^ Date that the power plant retired from using coal or oil as fuel source. Humboldt Bay and Port of Stockton later transitioned to burning biomass, but these transitions took place after our study period of interest (i.e., 0-12 months after the coal or oil retirement date).

^c^ Capacity of diesel fuel-fired unit.

^d^ Capacity for the entire power plant, which included generation from oil and natural gas.

**Table 2**: Change in median and interquartile range PM_2.5_ concentrations by area bin. Data in this table span 2002-2012 and come from the U.S. Environmental Protection Agency Community Multiscale Air Quality Model (CMAQ), available at <https://www.epa.gov/hesc/rsig-related-downloadable-data-files>. Because CMAQ data was unavailable prior to 2002, this table only includes data for 6 of the 8 power plants included in the main analysis, those that retired after 2003: Brawley, Humboldt Bay, Hunters Point, Port of Stockton, South Bay, and TXI Riverside.

|  | 12-24 months before retirement | | | | 0-12 months after retirement | | |
| --- | --- | --- | --- | --- | --- | --- | --- |
|  | **Area bin around power plant** | | | | **Area bin around power plant** | | |
|  | 0-5km | 5-10km | 10-20km | 0-5km | | 5-10km | 10-20km |
| **Annual average PM_2.5_ (μg/m^3^)**,  median (IQR) | 12.8  (9.0-17.4) | 12.3  (8.7-16.8) | 10.8  (7.7-15.5) | 11.5  (7.4-17.4) | | 11.0  (7.2-16.4) | 10.2  (6.4-14.3) |

**Table 3:** Analysis stratified by fuel type for the change in the annual fertility rate (live births per 1000 women aged 15-44 years) between 2001-2011 in California after 8 coal and oil power plant retirements.

|  | **Coal (n = 2 plants)*** | |  |  |  | **Oil (n = 6 plants)*** | |  |
| --- | --- | --- | --- | --- | --- | --- | --- | --- |
|  |  | **IRR (95% CI)^a^** | | |  |  | **IRR (95% CI)^a^** | |
| **Area bin^b^** | **Live births, N (%)*** | **Unadjusted** |  | **Adjusted** |  | **Live births, N (%)*** | **Unadjusted** | **Adjusted** |
| 0-5 km | 409 (0.7) | 1.19 (0.4-4.0) |  | 1.20 (1.19-1.25) |  | 8588 (14.5) | 1.2 (0.6-3.0) | 1.2 (1.0-1.4) |
| 5-10 km | 2819 (4.8) | 0.97 (0.3-4.5) |  | 0.99 (0.98-1.02) |  | 13650 (23.2) | 1.1 (0.6-2.3) | 1.1 (1.0-1.4) |

^a^ IRR obtained from difference-in-differences negative binomial regression model with robust standard errors, adjusted for power plant and proportion of births to Hispanic and non-Hispanic black mothers, mothers > 30 years of age, and mothers that attained a high school degree or less, and census block group level poverty (%), individuals with < high school education (%).

**^b^** 10-20km area bin served as the comparison population (it contained 33,443 live births).

* We note the small number of power plants in each group and the small numbers of births in these bins; for these reasons, we present combined coal and oil plants as our main analysis.

**Table 4:** Negative control analysis for the change in the annual fertility rate (live births per 1000 women aged 15-44 years) between 2001-2011 in California after 8 coal and oil power plant retirements.

|  | **Live births,**  **N (%)** | **Incidence rate ratio (IRR)**  IRR (95% CI)^a^ | | | |
| --- | --- | --- | --- | --- | --- |
| **Area bin^c^** |  | **Unadjusted** | **Adjusted^b^** |  |  |
| 0-5 km | 6264 (7.8) | 1.1 (0.8-1.4) | 1.1 (0.8-1.3) |  |  |
| 5-10 km | 14667 (18.3) | 1.1 (0.8-1.3) | 1.1 (0.9-1.3) |  |  |

^a^ IRR obtained from difference-in-differences negative binomial regression model with robust standard errors.

^b^ Adjusted for power plant and proportion of births to Hispanic and non-Hispanic black mothers, mothers > 30 years of age, and mothers that attained a high school degree or less, and census block group level poverty (%), individuals with < high school education (%).

**^c^** 10-20km area bin served as the comparison population (it contained 57,707 live births).

**Table 5:** Change in the annual fertility rate (live births per 1000 women aged 15-44 years) between 2001-2011 in California after 8 coal and oil power plant retirements, further adjusted for annual housing foreclosures

|  | **Live births,**  **N (%)** | **Annual fertility rate**  Mean (95% CI)^a^ | | | |
| --- | --- | --- | --- | --- | --- |
|  |  | **Unadjusted** | | **Adjusted^b^** | |
| **Area bin** |  | **1-2 years**  **before retirement** | **1 year**  **after retirement** | **1-2 years**  **before retirement** | **1 year**  **after retirement** |
| 0-5 km | 8997 (15.3) | 45 (28-61) | 53 (36-70) | 44 (34-54) | 51 (41-62) |
| 5-10 km | 16469 (28.0) | 43 (27-58) | 45 (29-61) | 45 (36-54) | 46 (37-56) |

^a^ Fertility rate obtained from difference-in-differences negative binomial regression model with robust standard errors.

^b^ Estimated at the mean value of covariates: power plant and proportion of births to Hispanic and non-Hispanic black mothers, mothers > 30 years of age, and mothers that attained a high school degree or less, and census block group level poverty (%), individuals with < high school education (%), and annual number of housing foreclosures.

**Table 6:** Demographic changes comparing before and after coal and oil power plant retirements in California, 2001-2011^a^

| **Demographics** | **Median (IQR) change** | | |
| --- | --- | --- | --- |
|  | 0-5km | 5-10km | >10km |
| Total population, number of residents | 2 (-3, 28) | 1 (-3, 17) | 0 (-2, 16) |
| Women, 15-50 years, number | 0 (-4, 0) | -1 (-4, 0) | -1 (-3, 0) |
| Non-Hispanic white, % | -1.8 (-4.3, -1.2) | -3.9 (-5.3, -1.8) | -2.1 (-2.7, -0.1) |
| Poverty, % | 0.2 (-0.1, 1.4) | 0.1 (0.2, 0.9) | 0.2 (-0.2, 1.1) |
| Median household income, $1000s | 21.8 (12.8, 28.9) | 24.0 (11.8, 31.2) | 21.2 (11.1, 32.4) |

^a^ Estimates based on data from the 2000 and 2010 censuses and the 2005-2009, 2006-2010, 2008-2012, 2009-2013, 2011-2015 American Community Survey (ACS). The closest census or ACS before and after were used to calculate changes.

We performed a sensitivity analysis to assess the change in the composition of individuals living near power plants before versus after retirement. Because block groups did not always fall within entirely a single buffer, we scaled population characteristics by the proportion of the block group area that fell within each buffer. For example, if 50% of block group A lay inside the 0-5km buffer and 50% lay within the 5-10km buffer, then we attributed 50% of block group A’s population to the 0-5km buffer and 50% to the 5-10km buffer. We standardized to the 2010 boundaries to account for changes in block group boundaries over time. We show these results in e**Table 6**.
